# Sex differences in the association between sarcopenia and mild cognitive impairment in the older Korean population

**DOI:** 10.1186/s12877-023-03911-4

**Published:** 2023-05-29

**Authors:** Hyo-jung Lee, Ju-Young Choi, Dongui Hong, Donghoon Kim, Jin-Young Min, Kyoung-Bok Min

**Affiliations:** 1 Department of Preventive Medicine, College of Medicine, Seoul National University, 103 Daehak-Ro, Jongno-Gu, Seoul, Republic of Korea; 2 Veterans Medical Research Institute, Veterans Health Service Medical Center, 53, Jinhwangdo-ro 61-gil, Gangdong-gu, Seoul, Republic of Korea; 3 Institute of Health Policy and Management, Medical Research Center, Seoul National University, 103 Daehak-Ro, Jongno-Gu, Seoul, Republic of Korea

**Keywords:** Aging, Sarcopenia, Gender difference, Cognitive function, Muscle mass

## Abstract

**Background:**

A link between sarcopenia and cognitive function has been proposed and is supported by several investigations. Nevertheless, the sex-linked relationship between these two diseases has been scarcely investigated. This cross-sectional study investigated sex differences in the association between sarcopenia and mild cognitive impairment.

**Methods:**

We included all 286 participants aged 60 years or older with MCI who visited the Department of Neurology at Veterans Health Service Medical Center in South Korea from January to December 2021. The diagnosis of MCI was confirmed by two neurologists based on the participants’ neuropsychological test scores. Diagnosis of sarcopenia was based on the algorithm of Asian Working Group for Sarcopenia (AWGS) 2019 including bioelectrical impedance analysis and handgrip strength, and cognitive function was assessed using Seoul Neuropsychological Screening Battery Core (SNSB-C) test.

**Results:**

Among the 286 participants, 171 and 112 were men and women. After adjustment for potential covariates including APOE genotype, in women participants, there were significant associations between diagnosis of sarcopenia and MCI (OR = 4.72, 95%CI [1.39–15.97]), while there was no significant relationship in men participants. In eight subdomains of SNSB-C, we also found that women participants with sarcopenia demonstrated a significant memory decline (OR = 3.21, 95%CI [1.01–10.19]) as compared with the reference women group without sarcopenia after adjusting all covariates mentioned above. No significant association between any SNSB-C subdomain and MCI was demonstrated in men participants.

**Conclusions:**

We demonstrated that there was a different relationship between sarcopenia and MCI by sex and that sarcopenia may affect the cognitive subdomain differently by sex. These results imply that, with regard to cognitive function, maintaining muscle function and muscle mass might be more crucial for women than for men.

## Introduction

The Asian Working Group for Sarcopenia (AWGS) Consensus 2019 defined sarcopenia as an age-related loss of muscle mass, low muscle strength, and/or low physical performance [1]. With the rapid increase in the population of adults aged 65 and over, the prevalence of sarcopenia is estimated to range from 9.9% to 40.4%, worldwide, depending on the definition used [2, 3]. Sarcopenia can diminish quality of life and lead to fall-related injuries, which increases the economic burden of medical care, including hospitalization. Recently, sarcopenia was awarded an International Classification of Diseases, Tenth Revision, Clinical Modification (ICD-10-CM) code (M62.84) and has recognized as a disease entity [4]. The etiopathogenesis of sarcopenia involves multifactorial processes, such as oxidative stress, inflammation, and changes in positive or negative regulators in the muscles. Among these contributory factors, aging-related sex hormonal deregulation is considered an important cause of sarcopenia [5]. A gradual decrease of androgens, estrogens, and progesterone by aging contributes to loss in muscular function and mass differently by sex [6, 7]. Likewise, serum IGF-1 demonstrated a protective effect for sarcopenia in women, but not in men [8]. The fact that the same hormone had a different effect on risk of sarcopenia on subjects of different sex implies that there are differential pathophysiological mechanisms for sarcopenia by sex.

A decline in cognitive function is generally expected with normal aging. Dementia, however, is a pathological, and not a physiological, age-related cognitive decline, and constitutes a syndrome that comprises brain disease with impairment of memory, thinking, orientation, calculation, learning capacity, language, and judgment. Dementia has been an enormous health burden in most countries and total payment of health care service for dementia patients are estimated to be $355 billion in 2021 in United States alone [9]; therefore, the early prediction and intervention of pathological cognitive function decline has become a serious issue. Mild cognitive impairment (MCI) has been considered a transition between normal cognition and dementia [10]. The prevalence of MCI among those aged 60 years or older varies widely in different countries, from 6.8% in Latin America to more than 15.5% in China [11–14]. In a longitudinal study, the conversion rate from MCI to dementia ranged from 10 to 15% per year, while age-matched control subjects developed dementia at a rate of 1% to 2% per year [15]. Interestingly, several studies have suggested that the incidence, prevalence, severity, and progression of cognitive impairment are affected by sex differences [16–18]. Age-related sex steroid hormone reduction increases the risk of cognitive decline because estrogens and androgens exert slightly different protective actions in neural functioning and vascularity [19]. For example, heart disease and myocardial infarction are risk factors for cognitive impairment only for men, not for women.

As sarcopenia and cognitive decline are common age-related diseases, a link between sarcopenia and increased risk of cognitive impairment has been actively studied and supported by several convincing meta-analysis [20, 21]. There is also plausible evidence that both diseases showed significant sex differences in clinical symptoms and that each disease is associated with sex hormones. Nevertheless, the sex-linked relationship between them has been scarcely investigated for its importance. Furthermore, which cognitive functions are significantly associated with each sex has remained unclear. We hypothesized that the association between sarcopenia and cognitive impairment would differ by sex and that the associations of each cognitive function subdomain and sarcopenia may also differ by sex. This finding would highlight the importance of sex-specific investigation of cognitive function and sarcopenia, and help plan different health-related interventions for each sex to prevent cognitive impairment.

## Methods

### Study population

Individuals aged ≥ 60 years who visited the Department of Neurology at the Veterans Health Service Medical Center (Seoul, Republic of Korea) between January and December 2021 were included for the current study. The inclusion criteria were as follows: 1) patients who complained of cognitive decline, 2) patients who could independently complete clinical tests and questionnaires, and 3) patients who voluntarily agreed to participate in this study. The exclusion criteria were: 1) a diagnosis of dementia (ICD-10: F00–F09 and G30); 2) diagnosis of brain infarction, cerebral hemorrhage, or Parkinson’s disease; and 3) presence of another serious disease (e.g., cancer or mental illness). The inclusion and exclusion criteria were evaluated by experienced neurological clinicians. The study protocol was approved by the Institutional Ethical Review Board of the Veterans Health Service Medical Center (IRB No. BOHUN 2021–02-024–001 and BOHUN 2021–01-066–006).

Participants underwent a health survey that consisted of gait measurements, cognitive and physical examinations, and questionnaires and was conducted at the Veterans Medical Research Institute of the Veterans Health Service Medical Center. A total of 575 individuals volunteered to participate in this study and provided informed consent at the time of enrollment. Of these, 276 participants subsequently dropped out of the study because of missing data on questionnaires (*n* = 117), physical examinations (*n* = 19), blood test (*n* = 133) and incomplete cognitive assessment due to personal cause (*n* = 7). By the result of the cognitive function test, 13 participants who were already diagnosed with dementia were excluded. The final study population comprised 286.

### Sarcopenia

Sarcopenia was diagnosed based on the AWGS algorithm [1]. In our clinical research setting, we screened the patients based on either calf circumference (< 34 and < 33 cm in men and women, respectively) or Strength, Assistance with walking, Rising from a chair, Climbing stairs, and Falls (SARC-F) questionnaire (≥ 4) to identify individuals who are at risk for sarcopenia. To confirm the diagnosis, low muscle strength was defined as a handgrip strength < 28 and < 18 kg for men and women, respectively, and we specified the criteria for low physical performance as a 6-m walk speed < 1.0 m/s. The cutoff for height-adjusted muscle mass was a bioimpedance of < 7.0 and < 5.7 kg/m^2^ in men and women, respectively. In our study, the SARC-F questionnaire was administered by a physician. The handgrip strength was measured three times for each hand, and the maximum grip strength was recorded as the participant’s muscle strength. The appendicular skeletal muscle mass (ASM), the sum of skeletal muscle mass of each limb, was estimated via multifrequency bioelectrical impedance analysis using InBody 370S (InBody, Seoul, South Korea). The appendicular skeletal mass index (ASMI) was calculated as the ASM/height^2 (ASM/m^2^).

### Neuropsychological test

In this study, the assessment of cognitive function included the Seoul Neuropsychological Screening Battery Core (SNSB-C). The Seoul Neuropsychological Screening Battery-Second Edition (SNSB-II) is a comprehensive neuropsychological test that is commonly used in South Korea. The SNSB-C is an alternative to SNSB-II because the sensitivity specificity and positive predictive value in diagnosing dementia is equivalent to that of SNSB-II, and the SNSB-C can be completed in a shorter duration (about 40 min) [22]. The SNSB-C consists of five cognitive domains: attention, language, visuospatial, memory, and frontal/executive [23]. Each domain consists of its own tests, such as Digit Span Test: Forward + Backward (DST: F + B) for the attention domain, Short-Korean version of the Boston Naming Test (S-K-BNT) for the language domain, Rey Complex Figure Test: Copy (RCFT) for the visuospatial domain, Seoul Verbal Learning Test: Delayed Recall (SVLT-DR) for the memory domain, Digit Symbol Coding (DSC), Trail Making Test-Elderly: Part B (TMT-E: B), Controlled Oral Word Association Test (COWAT), and Color Word Stroop Test: Color Reading (CWST) for the frontal/executive cognitive domain. The DST was measuring individual’s forward and backward immediate recall spans to assess attention with working memory. The S-K-BNT consists of 15 items and subjects should look at the picture presented and say its name. The RCFT copied the Rey complex figure devised by Andre Rey in 10 min. The SVLT was a test recalling the words 20 min after learning 12 items spoken by a tester. In DSC, the subjects were asked to write down symbols corresponding to each number as quickly as possible according to a reference table for two minutes. TMT-E: B was a test that alternately connects the numbers (1 through 15) and days of the week (Monday through Sunday). COWAT was a test to voluntarily report words starting with each semantic (animal) and phonemic (/g/) for one minute each. CWST was a test reading out 112 color words printed in a different color from its meaning for one minute as fast as possible. Subjects was diagnosed with cognitive decline when an score was below the 16^th^ percentile in each SNSB-C domain after comparing with age- and education-matched norms. This cut-off scores was derived from − 1 standard deviation (z-score ≤ -1.00) of the norm proposed by SNSB-C and SNSB [22]. The final diagnosis of dementia or MCI was confirmed by two neurologists based on the participants’ neuropsychological test scores.

### Other variables of interest

We considered demographics, health behavior, and medical history as the other variables of interest. Demographic variables that were obtained by on-site personal interviews included age (60–64, 65–69, 70–74, 75–79, and 80 + years), sex (men or women), monthly family income (< $20,000 or ≥ $20,000), current marital status (yes or no), and education level (less than high school graduation or college degree and higher). Physical activity was measured as the metabolic equivalent of task (MET) by using the International Physical Activity Questionnaire (IPAQ), which reflects the amount of physical activity per week. Other health behavior variables included self-reported smoking status (current smoker, former smoker, or never smoker), and current alcohol consumption (drinker or non-drinker). Medical histories were based on whether the patients had been diagnosed with hypertension (yes or no) or diabetes (yes or no) by a doctor. Disease histories of diabetes and hypertension were included as confounding variables because they were shown to be associated with a MCI and sarcopenia respectively in meta-analysis studies [14, 24]. Apolipoprotein E (APOE) genotype is known to be strongly associated with cognitive function [25], and therefore was included as a covariate (having allele 4 or other). Blood samples were taken from each participant in the research center and transferred to technicians in reputable institutions to test for the APOE genotype.

### Statistical analysis

All statistical analyses were performed using PROC procedures in SAS (version 9.4; SAS Institute, Cary, NC, USA). All tests were two-sided, and the level of statistical significance was set at α = 0.05. All variables, except age and METs, were treated as categorical variables in this study. For these categorical variables, the number of participants and percentages of the total number of participants were computed for each characteristic of the participants. Moreover, age and MET were treated as continuous variables and the mean and standard deviation were calculated. *P*-values for statistical significance are shown based on the chi-square test for categorical variables and the *Student’s t*-test for continuous variables.

To explore the association between sarcopenia and MCI by subdomain and sex, the participants were assigned to subgroups by their sex: men, and women. To assess the difference in the proportions between sexes, *p*-values for statistical significance were calculated using the chi-square test. Then, we performed unadjusted regression and adjusted logistic regression analyses to calculate the size of the intergroup difference between the sex-stratified groups with and without sarcopenia. We further conducted SNSB subdomain unadjusted regression and adjusted regression analysis. The adjusted model was adjusted for APOE 4 genotype, age, education, family income, physical activity, current marital status, alcohol consumption, smoking, and medical history (diabetes and hypertension). The composite scores of the SNSB-C were expressed as z-scores standardized for age, sex, and education. The score provides an index of overall cognitive functioning. It is an alternative to the Korean Mini-Mental State Examination (a brief global instrument used to assess cognitive abilities) for screening patients with cognitive impairment [26]. Individuals with MCI were defined as those with a percentile ≤ 16th in one or more sub-tests of the SNSB-C after comparing age-, sex-, and education-matched norms [27]. This cut-off score was derived from a − 1 standard deviation (z-score ≤ -1.00) of the norm proposed by SNSB-C and SNSB [19]. The final diagnosis of dementia or MCI was confirmed by two neurologists based on the participants’ neuropsychological test scores. Odds ratios (OR) and 95% confidence intervals (95% CI) were calculated. All variables, except age and MET, were treated as categorical variables when used as adjustment variables in the regression analysis.

## Results

### Participant characteristics

The sex-stratified characteristics of the study population are summarized in Table 1 as the number (N) and percentage (%) for categorical variables and the mean and standard deviation (SD) for continuous variables. Among the 286 participants, 171 and 112 were men and women, respectively. The mean age (SD) of the study population was 74.03 years (SD = 5.08). The proportion differed significantly by sex in regard to age, education level, current marital status, smoking status, alcohol consumption, history of hypertension, and diagnosis of sarcopenia. Men were more likely to be older (*p* < 0.0001), less educated (≤ 12 years vs. > 12 years; *p* = 0.001), married (married vs. unmarried or widowed; *p* < 0.0001), current smokers (lower than that in former smokers or never smokers, *p* < 0.0001), drinkers (at least 12 alcoholic drinks per year vs. less than 12 alcoholic drinks, *p* < 0.0001), more likely to have hypertension (yes vs. no, *p* = 0.0155), and more likely to have sarcopenia (yes vs. no, *p* = 0.0274). However, there were no sex-related differences in monthly income, history of diabetes, APOE genotype, or MET. The mean MET (SD) was 4008.3 (8852.9).

**Table 1:** Characteristics of subjects (*N*=286)

Characteristics	Total (N=286)		Men (N=171)		Women (N=115)		*p*-value
	N	(%)	N	(%)	N	(%)	
Age (year)							
60 ~ 64	12	(4.20%)	3	(1.75%)	9	(7.83%)	<0.0001
65 ~ 69	33	(11.5%)	7	(4.09%)	26	(22.6%)	
70 ~ 74	114	(39.8%)	73	(42.6%)	41	(35.6%)	
75 ~ 79	97	(33.9%)	70	(40.9%)	27	(23.4%)	
80 ~	30	(10.4%)	18	(10.5%)	12	(10.4%)	
mean (SD)	74.03	(5.08)	75.08	(4.35)	72.47	(5.67)	<0.0001
Education							
Under high school	220	(76.9%)	120	(70.1%)	100	(86.9%)	0.0010
Over college	66	(23.0%)	51	(29.8%)	15	(13.0%)	
Monthly income ($)							
≤ 20,000	128	(44.7%)	72	(42.1%)	56	(48.7%)	0.2718
> 20,000	158	(55.2%)	99	(57.8%)	59	(51.3%)	
Married (Current)							
Yes	243	(84.9%)	157	(91.8%)	86	(74.7%)	<0.0001
No	43	(15.0%)	14	(8.19%)	29	(25.2%)	
Smoking status							
Current smoker	13	(4.55%)	13	(7.60%)	0	(0.00%)	<0.0001
Ex-smoker	114	(39.8%)	112	(65.5%)	2	(1.74%)	
Never smoked	159	(55.5%)	46	(26.9%)	113	(98.2%)	
Alcohol drinking							
Drinker	95	(33.2%)	71	(41.5%)	24	(20.8%)	<0.0001
Ex-drinker	88	(30.7%)	71	(41.5%)	17	(14.7%)	
Non-drinker	103	(36.0%)	29	(16.9%)	74	(64.3%)	
History of diabetes							
Yes	70	(24.4%)	45	(26.3%)	25	(21.7%)	0.3774
No	216	(75.5%)	126	(73.6%)	90	(78.2%)	
History of hypertension							
Yes	176	(61.5%)	115	(67.2%)	61	(53.0%)	0.0155
No	110	(38.4%)	56	(32.7%)	54	(46.9%)	
Sarcopenia							
Yes	219	(76.6%)	138	(80.7%)	81	(70.4%)	0.0274
No	67	(23.4%)	33	(19.3%)	34	(29.6%)	
APOE genotype							
w/o allele 4	231	(80.7%)	136	(79.5%)	95	(82.6%)	0.5174
w/ allele 4	55	(19.2%)	35	(20.4%)	20	(17.3%)	
MET							
mean (SD)	4008.3	(8852.9)	4226.1	(9886.7)	3684.4	(7070.6)	0.5896

### Cognitive subdomains and sex

The number of participants with SNSB-C scores less than 16% by subdomain and sex, which is 1.0 SD below the mean proposed by SNSB, is shown in Table 2. The number (N) and percentage (%) of each sex and the *p*-value were calculated. Of the 286 participants, 189 (66.1%) were diagnosed with MCI. When the same participants were stratified by sex, 111 of 171 men participants and 78 of 115 women participants were diagnosed with MCI. However, the differences were not statistically significant. Additionally, the overall number of participants with an SNSB score less than 16% is shown in Table 2. Among the SNSB-C subdomains, the proportion differed significantly by sex in attention, language, memory, and frontal/executive tests. Women were likely to receive significantly lower scores on the attention test (*p* = 0.0327) and language test (*p* = 0.0035), whereas men were likely to receive lower scores on the memory test (*p* = 0.0006) and K-CWST-60:CR test (*p* = 0.0060).

**Table 2:** The number of participants with MCI or SNSB scores less than 16% by subdomain and sex

Score percentile of SNSB-C < 16%	Total (*N* = 286)		Men (*N* = 171)		Women (*N* = 115)		*p*-value
	N	(%)	N	(%)	N	(%)	
Diagnosis of MCI	189	(66.1%)	111	(64.9%)	78	(67.8%)	0.6098
SNSB-C							
Attention test (digit span)	66	(23.0%)	32	(18.7%)	34	(29.5%)	0.0327 *
Visuospatial test (RCFT)	34	(11.8%)	17	(9.94%)	17	(14.7%)	0.2187
Language (S-K-BNT)	33	(11.5%)	12	(7.01%)	21	(18.2%)	0.0035 *
Memory test (SVLT-DR)	101	(35.3%)	74	(43.2%)	27	(23.4%)	0.0006 *
Frontal/executive test							
Digit symbol Coding	33	(11.5%)	23	(13.4%)	10	(8.69%)	0.2172
COWAT: ㄱ + animal	64	(22.3%)	36	(21.0%)	28	(24.3%)	0.5121
TMT-E-PB	44	(15.3%)	24	(14.0%)	20	(17.3%)	0.4405
CWST-60:CR	69	(24.1%)	51	(29.8%)	18	(15.6%)	0.0060 *

*SNSB-C* Seoul Neuropsychological Screening Battery Core, *RCFT* Rey Complex Figure Test, *S-K-BNT* Short-Korean version of Boston Naming Test, *SVLT-DR* Seoul Verbal Learning Test-Delayed Recall, *COWAT* Controlled Oral Word Association Test, *TMT-E-PB* Trail Making Test-Elderly: Part B, *CWST* Color Word Stroop Test

^*^p < 0.05

### Association by sex with sarcopenia or MCI

**Fig. 1:**
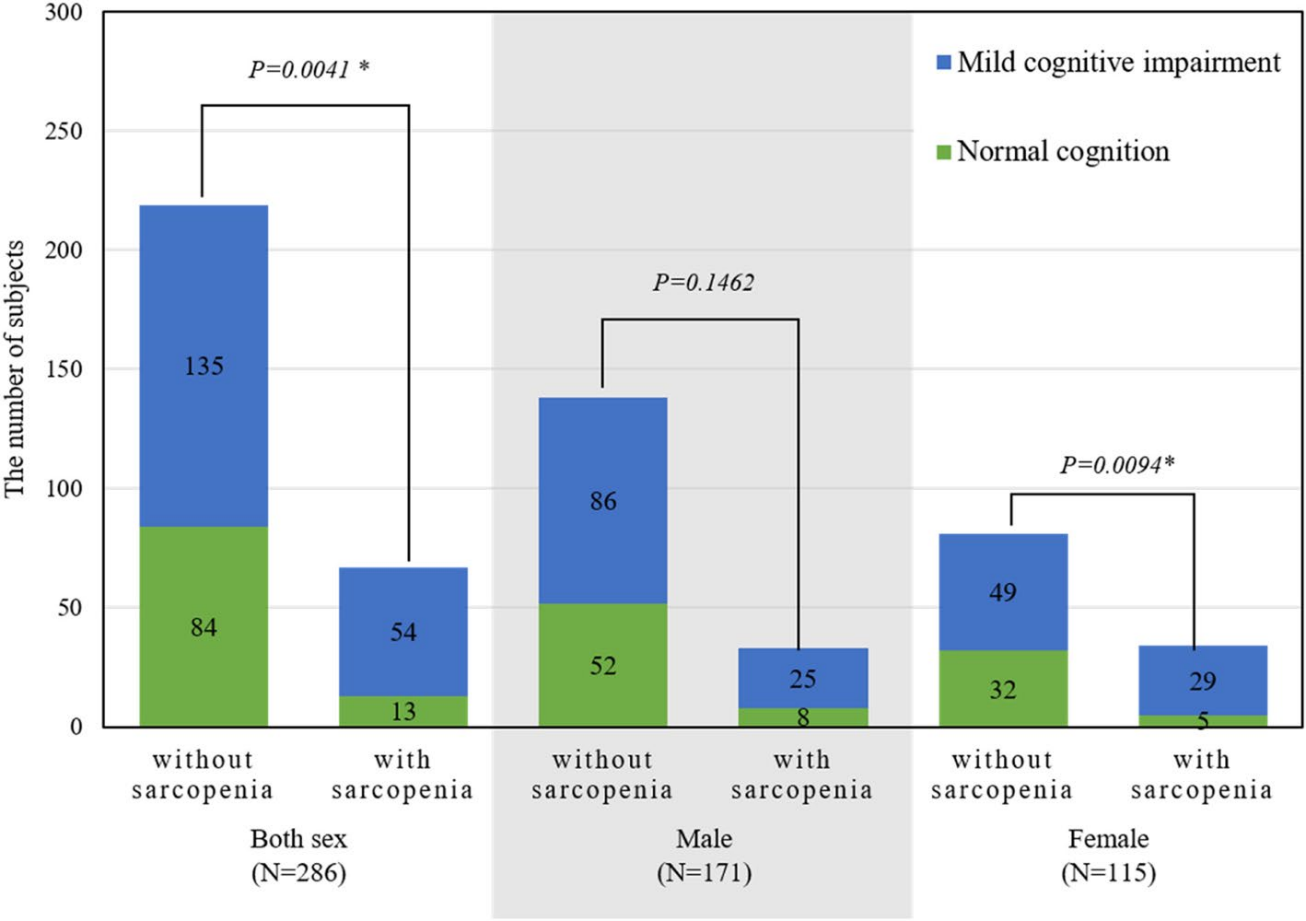
The association between sarcopenia and MCI according to sex

Figure 1 shows the number of participants with sarcopenia or MCI according to sex. Among the 286 participants, 54 simultaneously had both sarcopenia and MCI, whereas 84 had neither. The association between the diagnosis of sarcopenia and MCI was significant (*p* = 0.0041). When divided according to sex, the strength of the association between the two diseases differed. There were 25 men participants with sarcopenia and MCI and 52 men participants without sarcopenia or MCI, which suggested the absence of a significant association. However, in the women group, the association between sarcopenia and MCI was significant (*p* = 0.0094), as 29 women participants had both diseases.

### Regression analysis of association 

Table 3 shows the results of the multivariate analysis of the association between MCI diagnosis and sarcopenia. The ORs and 95% CI were calculated using logistic regression models. Participants without sarcopenia were used as the reference group, and participants who met the diagnostic criteria for sarcopenia were included in the sarcopenia group. We analyzed data using two regression models: an unadjusted regression model and a model that was fully adjusted for age, sex, the presence of APOE 4 allele, and demographic factors, such as education level, family income, physical activity, marriage, health behavior (alcohol and smoking), and medical history (diabetes and hypertension). The unadjusted linear regression model showed a positive relationship between the diagnosis of sarcopenia and MCI (OR = 2.59, 95% CI [1.33–5.02]) for both sexes. After adjusting for other covariates, the positive association persisted for both sexes (OR = 1.89, 95% CI [1.23–5.05]). When stratified by sex, no significant relationship between the diagnosis of sarcopenia and MCI was demonstrated in men participants. However, in women participants, we found a stronger association between the diagnosis of sarcopenia and MCI with the unadjusted models (OR = 3.79, 95% CI [1.33–10.81]) and the adjusted models (OR = 4.72, 95% CI [1.39–15.97]).

**Table 3:** Regression analysis of the association between sarcopenia, MCI, and SNSB score less than 16% by sex

	Both (*N* = 286)				Men (*N* = 171)				Women (*N* = 115)			
	Unadjusted		Adjusted ^a^		Unadjusted		Adjusted ^a^		Unadjusted		Adjusted ^a^	
	OR	(95% CI)	OR	(95% CI)	OR	(95% CI)	OR	(95% CI)	OR	(95% CI)	OR	(95% CI)
Diagnosis of MCI	2.59	(1.33, 5.02)*	2.49	(1.23, 5.05)*	1.89	(0.79, 4.45)	1.50	(0.57, 3.96)	3.79	(1.33, 10.81)*	4.72	(1.39, 15.97)*
SNSB-C												
Attention test (digit span)	1.45	(0.78, 2.70)	1.33	(0.67, 2.645)	1.22	(0.47, 3.12)	1.19	(0.43, 3.26)	1.46	(0.62, 3.44)	1.20	(0.418, 3.47)
Visuospatial test (RCFT)	1.43	(0.64, 3.16)	0.96	(0.40, 2.32)	0.53	(0.11, 2.44)	0.28	(0.05, 1.58)	2.46	(0.85, 7.05)	2.39	(0.575, 9.92)
Language (S-K-BNT)	1.05	(0.45, 2.46)	0.98	(0.36, 2.62)	2.24	(0.63, 7.95)	3.12	(0.61, 15.83)	0.50	(0.15, 1.62)	0.32	(0.07, 1.41)
Memory test (SVLT-DR)	1.56	(0.89, 2.73)	1.80	(0.97, 3.36)	1.51	(0.70, 3.24)	1.52	(0.67, 3.45)	2.40	(0.97, 5.90)	3.21	(1.01, 10.19)*
Frontal/executive test												
Digit symbol	1.26	(0.56, 2.86)	1.45	(0.59, 3.56)	1.19	(0.40, 3.48)	1.49	(0.46, 4.81)	1.67	(0.43, 6.32)	2.32	(0.40, 13.26)
COWAT: ㄱ + animal	2.06	(1.12, 3.80)*	1.33	(0.69, 2.53)	1.26	(0.51, 3.09)	0.90	(0.36, 2.24)	3.35	(1.37, 8.18)*	1.74	(0.59, 5.08)
TMT-E-PB	1.46	(0.72, 2.99)	0.93	(0.42, 2.07)	0.81	(0.25, 2.57)	0.55	(0.16, 1.92)	2.29	(0.84, 6.17)	1.25	(0.36, 4.34)
CWST-60:CR	1.09	(0.58, 2.06)	1.28	(0.63, 2.59)	0.86	(0.36, 2.00)	0.90	(0.35, 2.33)	2.19	(0.77, 6.13)	1.65	(0.456, 5.93)

*Notes*: ^a^This Model is adjusted for APOE 4 genotype, age, education, family income, physical activity, and marriage, alcohol, smoking and disease history (diabetes and hypertension), *SNSB-C* Seoul Neuropsychological Screening Battery Core, *RCFT* Rey Complex Figure Test, *S-K-BNT* Short-Korean version of Boston Naming Test, *SVLT-DR* Seoul Verbal Learning Test-Delayed Recall, *COWAT* Controlled Oral Word Association Test, *TMT-E-PB* Trail Making Test-Elderly: Part B, *CWST* Color Word Stroop Test

^*^ *p* < 0.05

Table 3 also shows the association between the diagnosis of sarcopenia and impaired subdomain function by sex. More detailed cognitive function analysis results are presented for the SNSB-C sector. Cognitive subdomain impairment was diagnosed when the score of each subdomain was less than 16^th^ percentile. For both sexes, participants with sarcopenia had a marginally higher significant chance of memory function decline (OR = 1.80, 95% CI [0.97–3.36]) compared with the reference group without sarcopenia after adjusting for covariates, including APOE 4 gene, age, education, family income, physical activity, current marital status, alcohol consumption, smoking, and medical history. Moreover, women participants with sarcopenia demonstrated a significant memory decline (OR = 3.21, 95% CI [1.01–10.19]) as compared with the reference women group without sarcopenia after adjusting for all of the abovementioned covariates. Other cognitive function tests did not demonstrate any relationship with the different sarcopenia groups in the adjusted models.

## Discussion

### Main findings

In this cross-sectional study, sarcopenia was significantly associated with a decline in cognitive function in women adults aged 60 + years in South Korea after adjusting for covariates, including the APOE4 allele. Especially in the SNSB subtest, women with sarcopenia were significantly more likely to have a decline in memory function than women without sarcopenia. These results suggest that sarcopenia might be more associated with cognitive function decline for women than for men. This finding implies that avoiding sarcopenia in women may be helpful for preventing cognitive impairment, especially with regard to memory function.

### Sarcopenia and cognitive impairment

First, our finding of an association between sarcopenia and comprehensive cognitive function decline is consistent with previous studies. With regard to this association, a Korean study conducted among community-dwelling Korean older women found that the MMSE score was negatively and linearly associated with AWGA-based sarcopenia severity [26]. Another meta-analysis with 5994 participants showed that the crude OR was 2.926 (95% CI 2.297–3.728) and the adjusted OR, which was adjusted for age, sex, education, depression, activities of daily life, and physical performance, was 2.246 (95% CI, 1.210–4.168) [21]. For the association between sarcopenia and MCI only, few studies have focused on the association between MCI and sarcopenia. In cross-sectional studies, a study conducted among outpatients with diabetes in Japan showed adjusted ORs of 2.96 (95% CI 1.09–7.70) and the other study in China showed OR of 1.67 (95% CI 1.04–2.68) for the sarcopenic group when compared to the non-sarcopenic control group [11, 28]. This association can be attributed to several physiological factors such as nutritional restriction and physical capacity impairment [29, 30] or common biological pathways, such as inflammatory cytokines such as interleukin-6 (IL-6) and interleukin-8 (IL-8) which are secreted in the skeletal muscle. These cytokines are thought to play a role in neuronal differentiation, leading to a decline in cognitive function [31]. Additionally, predisposing factors underlying sarcopenia may have been common risk factors for cognitive impairment, such as obesity, oxidative stress, cerebrovascular disease, disease, and insulin resistance [32, 33]. A recent prospective study revealed that sarcopenia was an independent risk factor of cognitive decline for older adults aged 65 and older (sarcopenia group vs. normal group: OR = 7.86, 95% CI = [1.53–40.5]) [34]. More prospective studies are needed to explore the causality between sarcopenia and cognitive function [35].

### Sarcopenia and memory function

Furthermore, we performed a subdomain analysis to assess which cognitive domain is associated with sarcopenia. Based on the results of this study, we observed that sarcopenia was associated with overall cognitive function decline and was not significantly associated with any subdomain, except that of memory function in women. Unfortunately, no studies have directly studied this topic; however, this link between sarcopenia and memory function has been implied by the association between physical activity and memory-specific function. In 2009, a 6-month randomized controlled trial study among women aged 70–80 years in Canada concluded that the regular physical activity group remembered significantly more items in the verbal memory test (*p* = 0.04) and that there was a significant correlation between spatial memory performance and overall physical capacity after intervention in the aerobic training group [36]. In another study that used magnetic resonance imaging in 165 non-dementia older adults, aerobic fitness was related to larger hippocampal volume and better spatial memory performance [37].

### Sex differences

Interestingly, however, no studies have focused on sex differences in regard to the association between sarcopenia and cognitive function despite the considerable influence of sex hormones. In our study, sarcopenia was not associated with cognitive impairment in older men, but was significantly associated with cognitive impairment in older women. These differences are supported by distinctions in gender physiology. Sex hormones affect sarcopenia. A meta-analysis that addressed the effectiveness of androgen treatment on muscle strength in men aged ≥ 65 years demonstrated a medium treatment effect [38]. They found that participants receiving testosterone/DHT treatment performed better in terms of overall muscle strength than the control group (19.3%). Likewise, sex hormones, such as DHEAS and testosterone, can affect muscle mass in both sexes through protein synthesis and cognitive function through nerve regeneration [5]. Furthermore, Sex differences, induce differences in hippocampal plasticity, activation, and morphology because men and women have a different distribution of sex hormone receptors in the hippocampus, such as androgen receptors and estrogen receptors [39]. Therefore, sex differences can affect neurogenesis and hippocampus-dependent strategies, suggesting that sex hormones are closely related to both sarcopenia and cognitive function and make the differences. Estradiol, a hormone secreted more in women, also plays a key role in sex differences. Similar to that of testosterone, the role of estradiol in sarcopenia has been well-demonstrated. Estradiol has a beneficial effect on skeletal muscle by stimulating satellite cell proliferation through receptors at the level of muscle fibers, and by controlling the inflammation level in skeletal muscle [40]. In a longitudinal community cohort, menopause was demonstrated to be related to a decrease in immediate and delayed recall (*p* = 0.03) [41]. Furthermore, the effects of estrogen on memory decline have been suggested in surgically menopausal women who have undergone hysterectomy and bilateral oophorectomy [42]. After menopause, a change in endocrine function derived from estrogen, FSH, DHEA, GH, IGF-1, and insulin had a negative influence on muscle mass; Type II fiber and motor units decreased while intramuscular fat increased [43].

In this study, we observed the gender-related association between sarcopenia and cognitive function. In the cognitive tests, including attention, language, memory, and frontal/executive tests, women and men scored lower in different subdomains, respectively. Women were likely to receive significantly lower scores on the attention and language tests, whereas men were likely to receive lower scores on the memory test and K-CWST-60:CR test. Also, sarcopenia appeared to have a more significant impact on women than on men. Although there were no significant differences in each subdomain of cognitive tests, women with sarcopenia were diagnosed more with MCI than those without sarcopenia. So far, several investigations have found an association between sarcopenia and cognitive function. The association between sarcopenia and memory function has been relatively straightforward, but no studies have examined gender differences. This result suggests that sarcopenia may affect cognitive function differently across genders.

### Strength and limitations

To the best of our knowledge, this is the first study to suggest sex differences in the association between sarcopenia and cognitive function. This study is valuable because sex differences have been understudied for their importance. To mitigate gene effects, we adjusted the APOE4 genotype in the presented models. Even after adjusting for possible covariates, this association remained statistically significant. However, our study had a few limitations. First, because this was a cross-sectional study, we cannot guarantee temporality and causality. The second limitation was the possibility of selection bias and applicability to the general population. We selected study participants from the outpatients at the Veterans’ Hospital for retired soldiers and their families. Old veterans included those who were wounded in the Korean War and Vietnam War and old veterans’ status tends to be relatively lower than general population [44, 45]. Outpatient clinic doctors tried to randomly select study participants from visitors who were suspected of having cognitive function decline; however, it cannot be ruled out that an injury suffered decades ago or other health disease may have affected cognitive function. Therefore, this result may not be applicable to the general population. This study attempted to reduce this limitation by adjusting for possible covariates including demographic factors, health-related variables, and disease history. Nevertheless, we cannot exclude residual confounding due to unconfirmed disease history or other health-related variables (such as hormonal dysregulation) that may affect cognitive function. Additionally, the biological explanation of the relationship between sarcopenia and cognitive function has still not been sufficiently presented. Finally, measurement errors can occur. Although the SNSB-C test was conducted by experienced experts in the same space for every participant, the test score may have differed because of human errors.

## Conclusions

This study clearly showed significant sex differences in the association between sarcopenia and cognitive function. Furthermore, by analyzing the overall cognitive function assessment, it was possible to demonstrate which cognitive domain's function was individually associated with sarcopenia, which is definitely a memory function. Although further large-population based research to clarify the biological explanation is inevitable, this finding suggests that sarcopenia may affect cognitive function differently by sex. Given the importance of this result, it may help understand the importance of sex-specific investigation of cognitive function and sarcopenia in research, and help consider sex-specific intervention for women to preventive cognitive impairment in practice.

## Data Availability

The datasets generated and analysed during the current study are not publicly available due to institutional restrictions but are available from the corresponding author on reasonable request.

## Abbreviations

AD: Alzheimer's disease
APOE: Apolipoprotein E
AWGS: Asian Working Group for Sarcopenia
CI: Confidence interval
COWAT: Controlled Oral Word Association Test
CWST: Color Word Stroop Test
DSC: Digit Symbol Coding
DST: F + B: Digit Span Test: Forward + Backward
MCI: Mild Cognitive Impairment
MET: Metabolic equivalent of task
RCFT: Rey Complex Figure Test
SARC-F: Strength, Assistance with walking, Rising from a chair, Climbing stairs, and Falls
SD: Standard deviation
SNSB-C: Seoul Neuropsychological Screening Battery Core
S-K-BNT: Short-Korean version of Boston Naming Test
SVLT-DR: Seoul Verbal Learning Test-Delayed Recall
TMT-E:B: Trail Making Test-Elderly: Part B
